# Supplementary material for: The effects of ground-irregularity-cancelling prosthesis control on balance over uneven surfaces
Source: R Soc Open Sci. 2021 Jan 20;8(1):201235. doi: 10.1098/rsos.201235 (PMC7890502; doi:10.1098/rsos.201235)
Supplement: Supplementary Text [file rsos201235supp1.pdf]

Supplementary Text

1 Terrain profile simulation

The unraveled treadmill belt measures 320 cm (126 in) long and 56 cm (22 in) wide. We discretized the terrain pattern into a 126x22 matrix with the value at each index equal to the height of the treadmill at that location. We then evaluated the sagittal and frontal angles of the prosthesis at every location in the matrix to form a histogram of disturbance angles for that terrain profile.

Fig. 1 shows four candidate patterns and their corresponding histograms. The Random profile consists of random heights at each index. This profile does not have a very uniform disturbance distribution and is extremely difficult to fabricate due to its complexity. The Random Blocks profile uses random heights in larger blocks and results in more uniform disturbances. The Stripes profile uses a repeating block pattern which improves ease of fabrication due to repeating structures, but does not have a very uniform disturbance distribution. The Waves profile offers a good balance between disturbance distribution and ease of fabrication and was the profile we chose to build.

2 Foot angle while walking on terrain

To demonstrate the terrain pattern is aperiodic, we calculated the sagittal ankle angle when the shank was vertical on each stride. Fig. 2 shows a representative trial of a participant walking on uneven terrain using the irregularity-cancelling controller. We do not have corresponding data for level ground since the participants only used their prescribed prosthesis for those trials.

3 Table of Non-Variable Control Parameters

Table 1 shows a list of prosthesis parameters set during the fitting session prior to the experiment. These parameters were kept constant throughout the experiment.

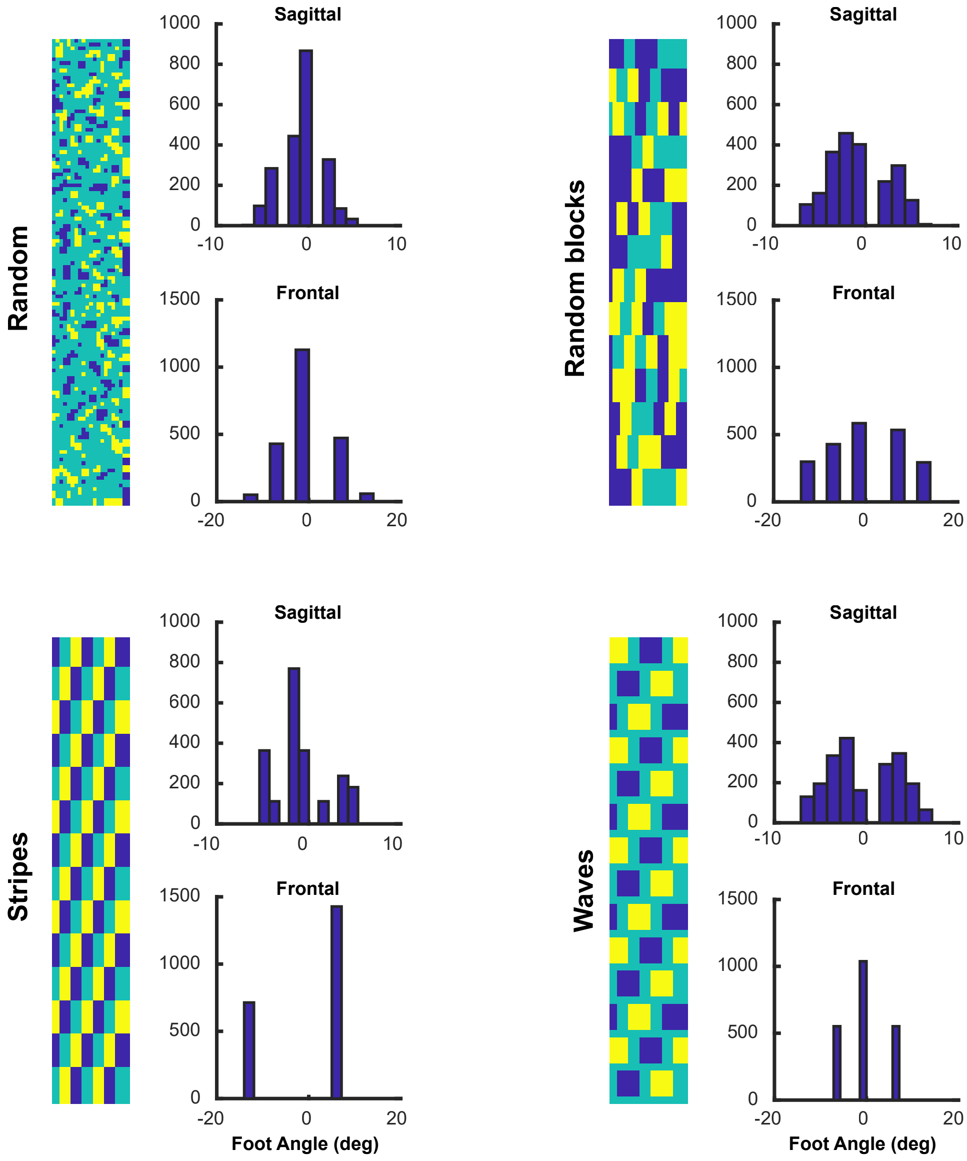

Figure 1: Four candidate terrain profiles and associated disturbance angle histograms

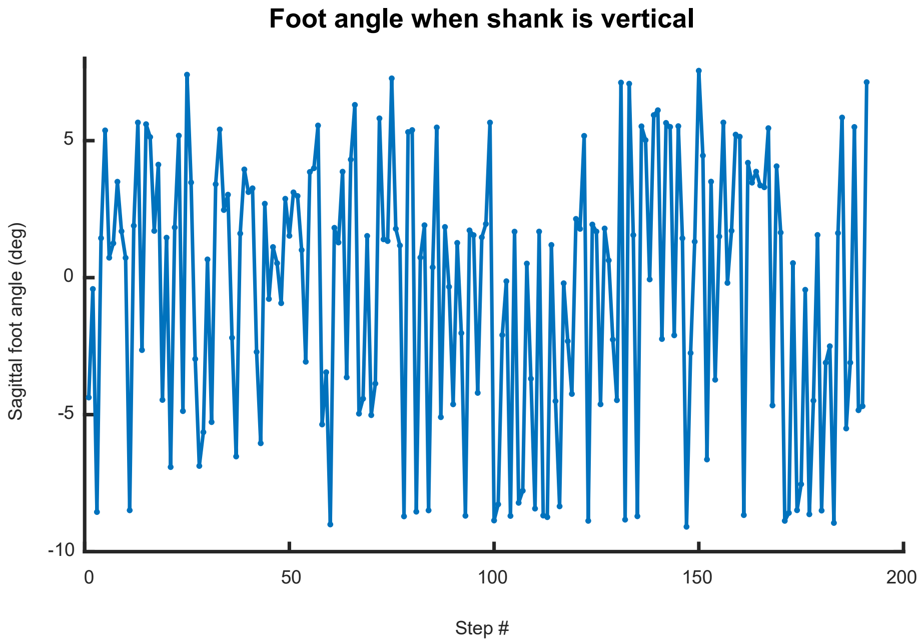

Figure 2: A representative walking trial shows the terrain results in aperiodic foot angle

Table 1: Non-Variable Control Parameters

Participant	Forefoot Stiffness (Nm/rad)	Heel Stiffness (Nm/rad)	Heel Neutral Angle (rad)	Plantarflexion Velocity (rad/s)	IMU Threshold Angle (deg)
1	300	250	2.1	20	0
2	300	200	1.9	20	-6.5
3	300	450	2.1	15	-4
4	200	175	1.9	25	0
5	250	300	1.9	20	-6
