# Supplementary material for: The effects of ground-irregularity-cancelling prosthesis control on balance over uneven surfaces
Source: R Soc Open Sci. 2021 Jan 20;8(1):201235. doi: 10.1098/rsos.201235 (PMC7890502; doi:10.1098/rsos.201235)

Treadmill Modification Tips

This document is intended to provide tips and general guidance on attaching an uneven surface to a commercially-available treadmill. In our case, we modified a Horizon Elite T9 treadmill.

Disassembly

In order to attach blocks onto the treadmill belt, we disassembled the treadmill until we were able to access and remove the treadmill deck, belt, and rollers. The image below shows the treadmill with exposed deck and rollers after its plastic cladding has been removed.

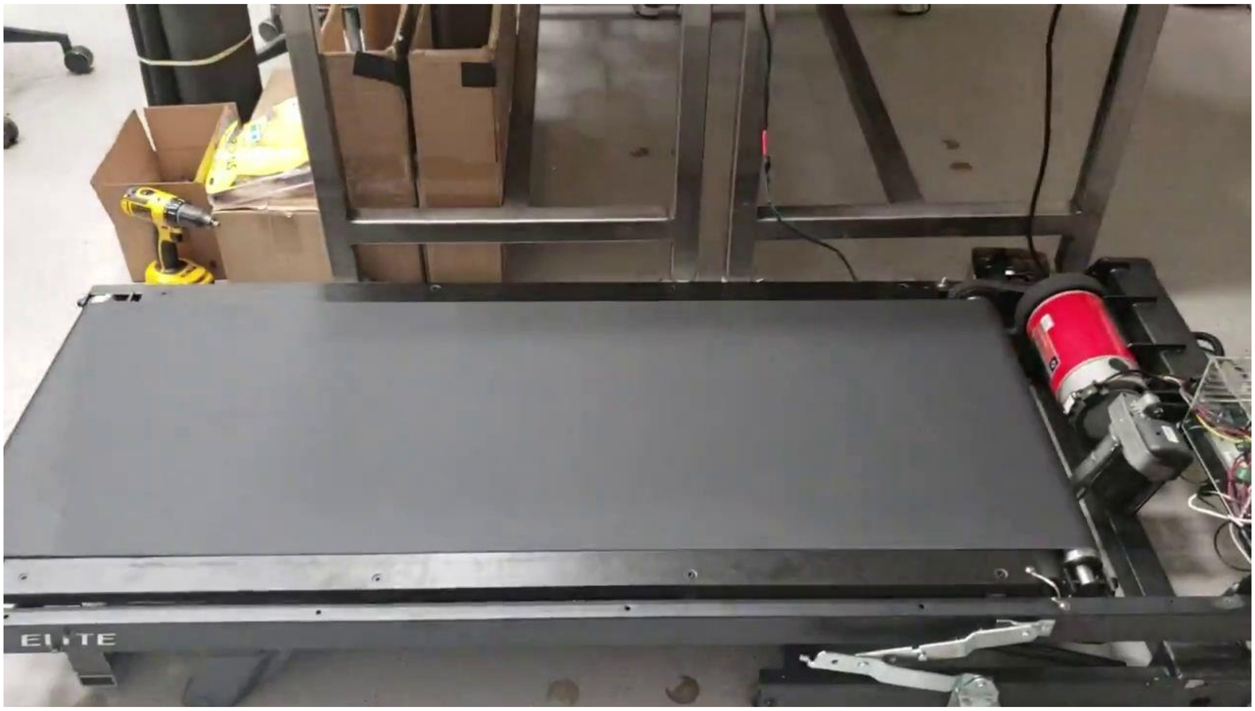

Relocating the Control Panel

The treadmill control panel, including the screen and buttons, were cut from the factory panel and combined into a more compact format. Unneeded electronics such as the built-in fan or heart rate monitor were discarded. We extended the wire leads between the control box and the treadmill in order to control the treadmill from the experimenter’s desk. The pull-cord emergency stop was replaced with a toggle switch located at the bottom right of the panel. The image below shows the compact control panel, keeping only the screen, speed and incline buttons, and the emergency stop switch.

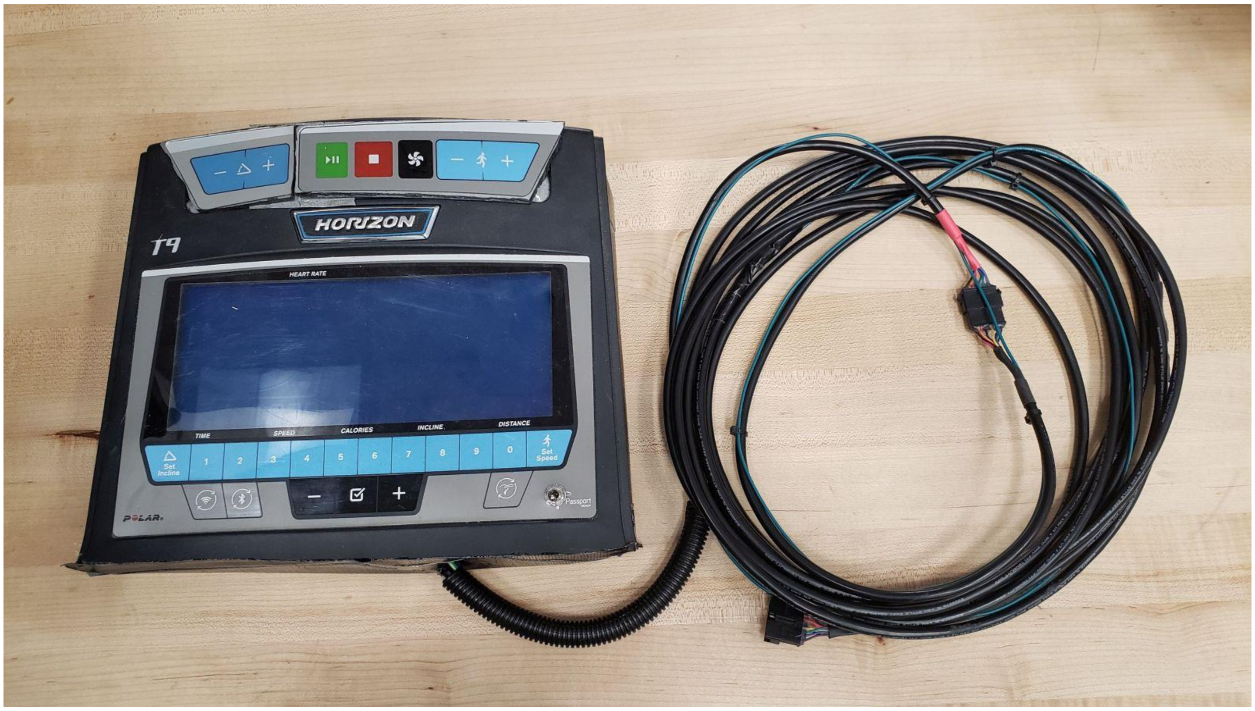

Providing Clearance for Wooden Blocks

The added height of the wooden blocks interfered with two frame crossbars as well as the motor drive electronics. We cut and replaced the factory crossbars with custom U-shaped bars to allow for additional clearance. We used L-brackets (McMaster #11765A12) in our crossbar design. We also relocated the motor drive electronics to the front of the treadmill.

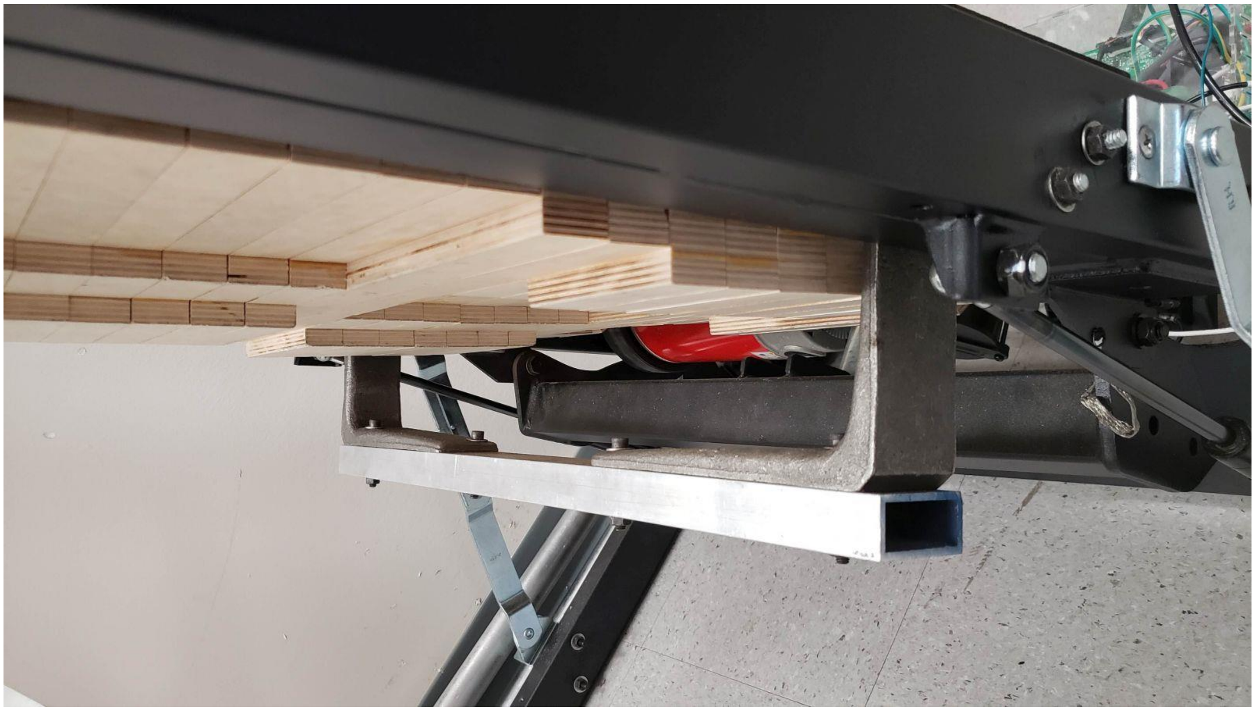

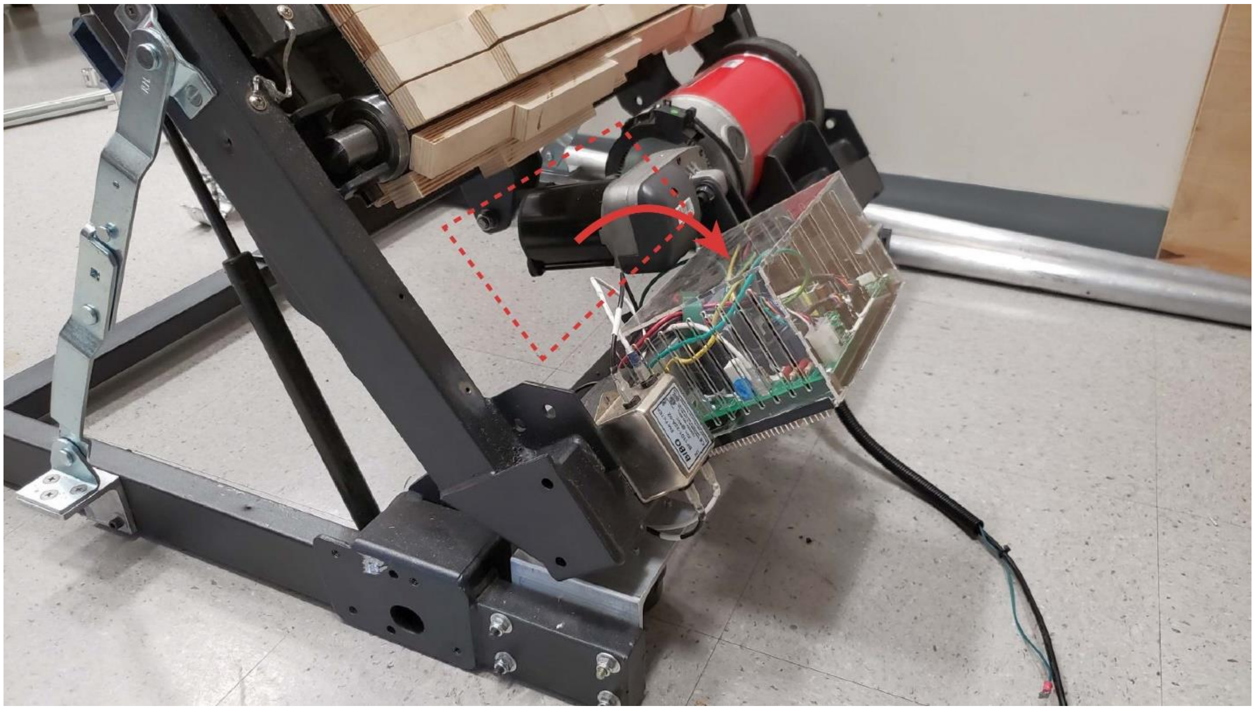

Fabricating Wooden Blocks

We used Baltic Birch plywood sourced from https://www.woodworkerssource.com/. We chose plywood since it is available in convenient 1/4” increments and we chose Baltic Birch plywood due to its greater strength compared to other types of plywood.

To streamline fabrication, we first glued pre-cut sheets of plywood together before cutting into 1” strips. For example, the blocks with color annotations in the picture below were created by gluing together three sheets of plywood: 22” x 14+” x 1/4” (green), 12” x 14+” x 1/2” (red), and 6” x 14+” x 1/2” (blue). The lengths are listed as 14+” as a reminder to compensate for the kerf width of the saw blade.

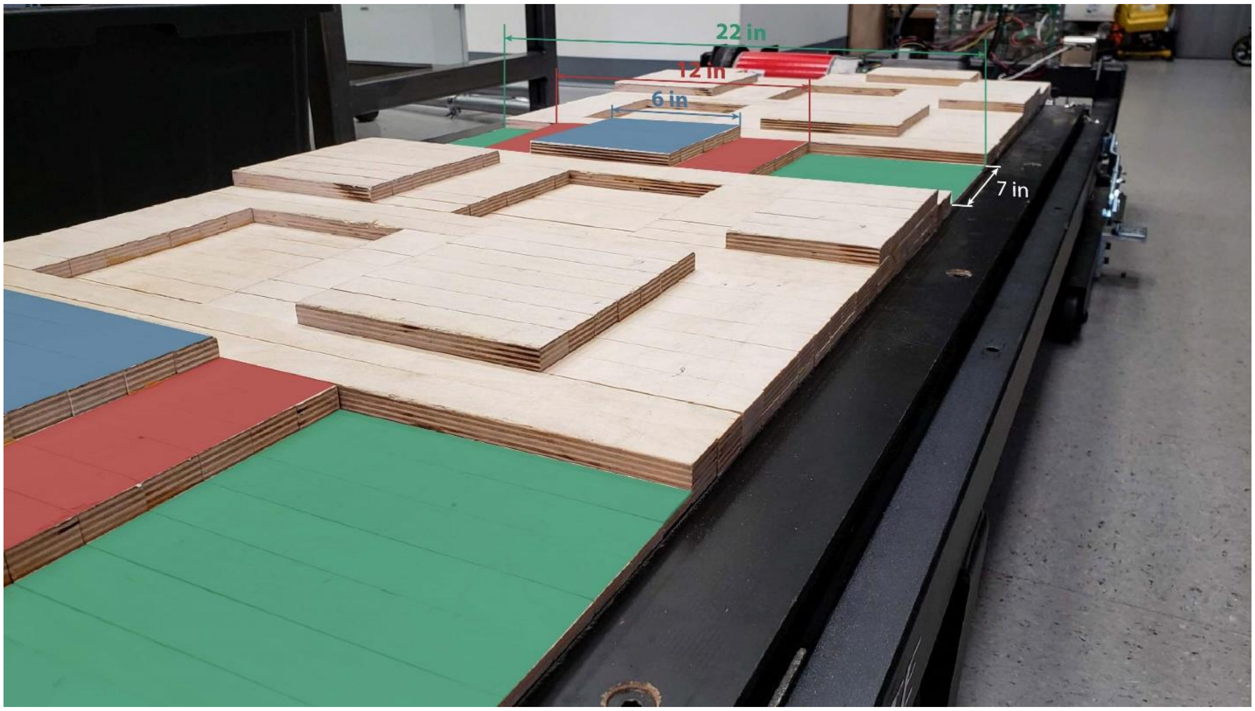

Attaching Wooden Blocks

To attach the blocks onto the treadmill, we stapled the blocks onto belt from the underside of the belt using 3/8” staples at three locations per block. The 80/20 frame shown in the picture below was built to make stapling easier. Depending on the strength of your staple gun, you may need to hammer the staples after stapling so that they sit flush or inset into the belt such that the staple heads do not drag across the treadmill deck. Be sure to wear eye and ear protection!

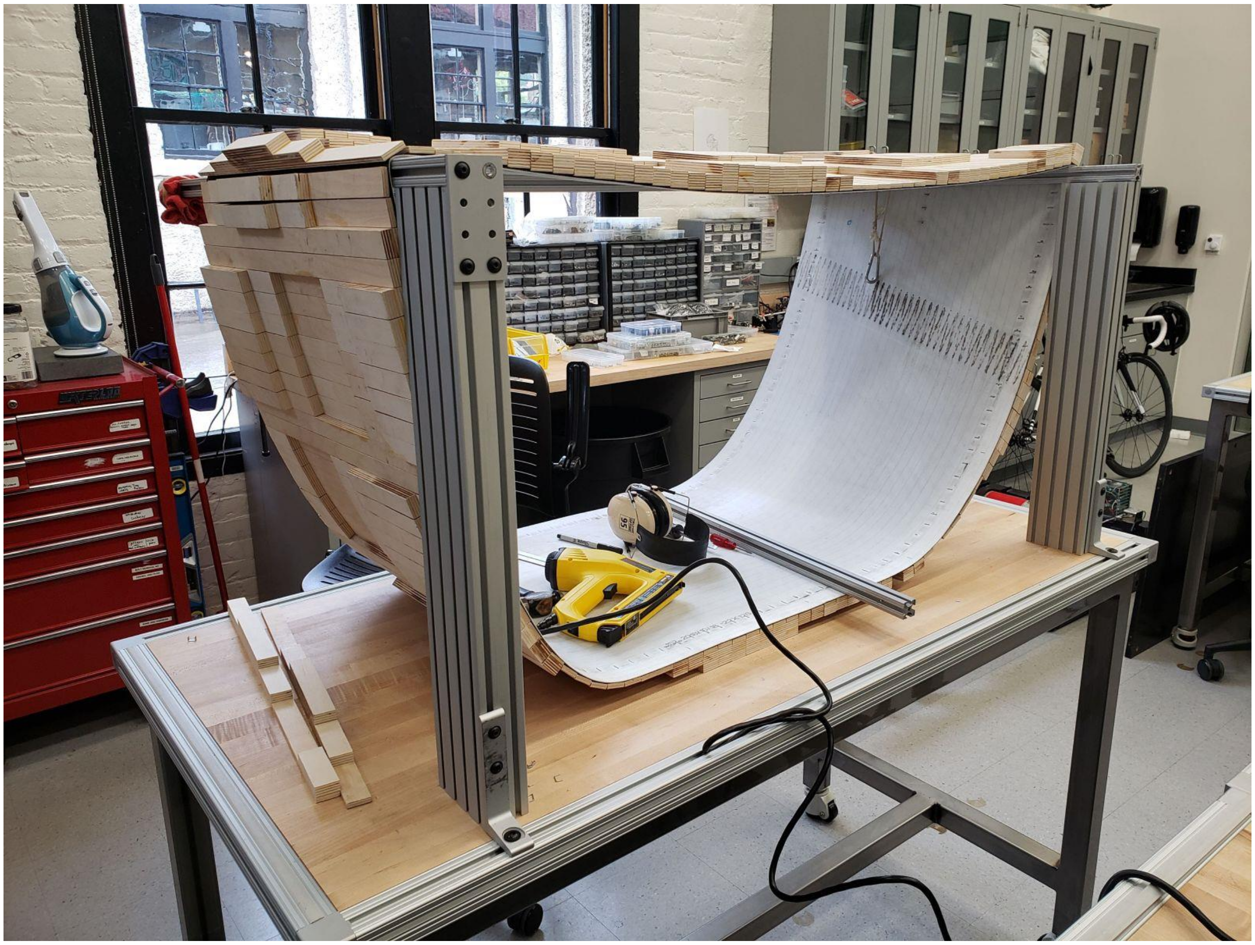

Miscellaneous

Remember to lubricate the treadmill deck with silicone oil as you reassemble the treadmill. During reassembly, you will also need to align the treadmill belt by adjusting the rollers. Information on the alignment procedure is readily available online. This video shows the adjustment procedure for the rear roller.

We removed the built-in handrails during disassembly and instead built an external set of handrails located in front of and on both sides of the participant. The handrails are clamped onto an existing set of handrails used for the level-ground treadmill.

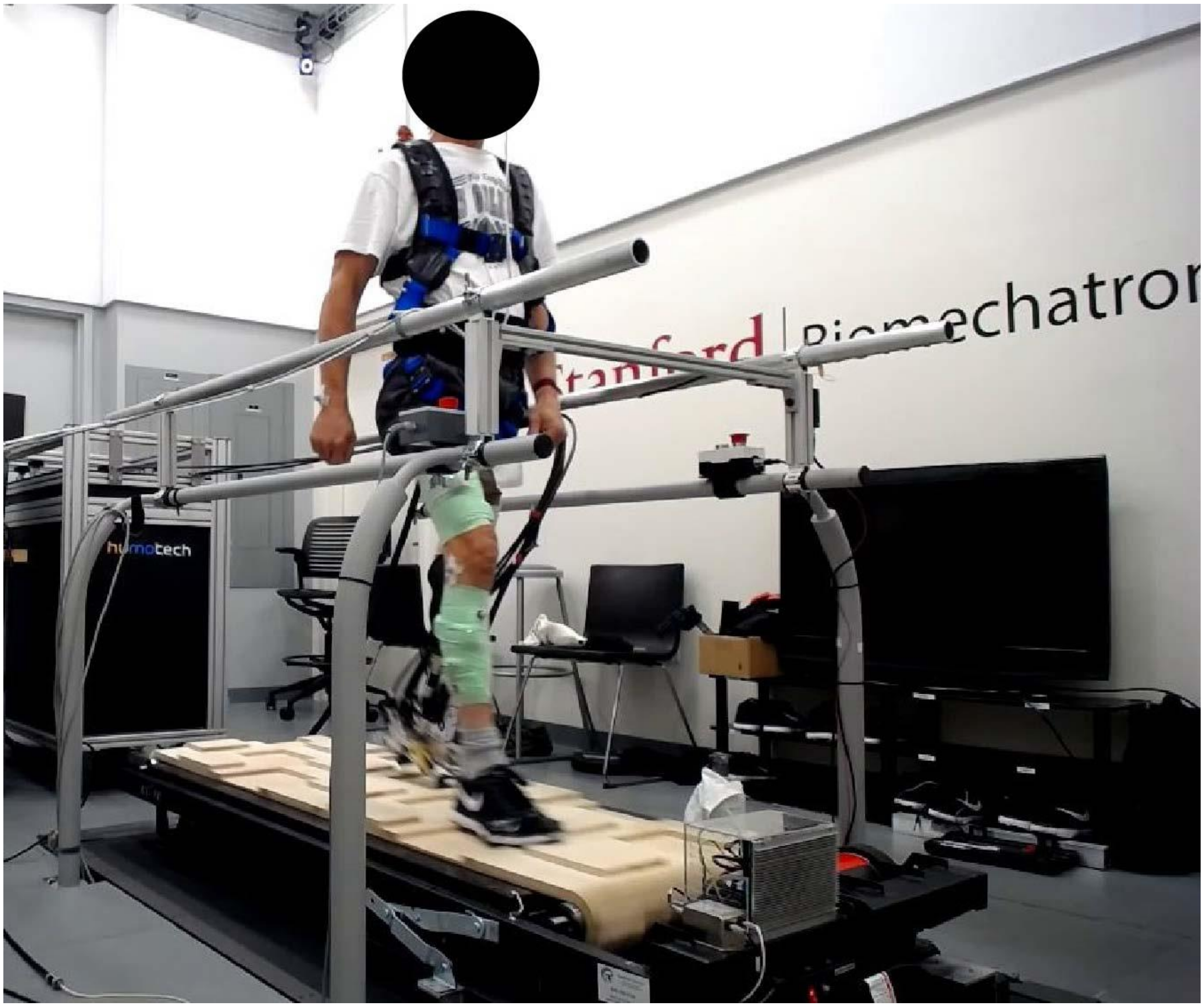

We installed two caster wheels (McMaster #24125T23) onto the front of the treadmill for improved maneuverability. The front left wheel is shown below.

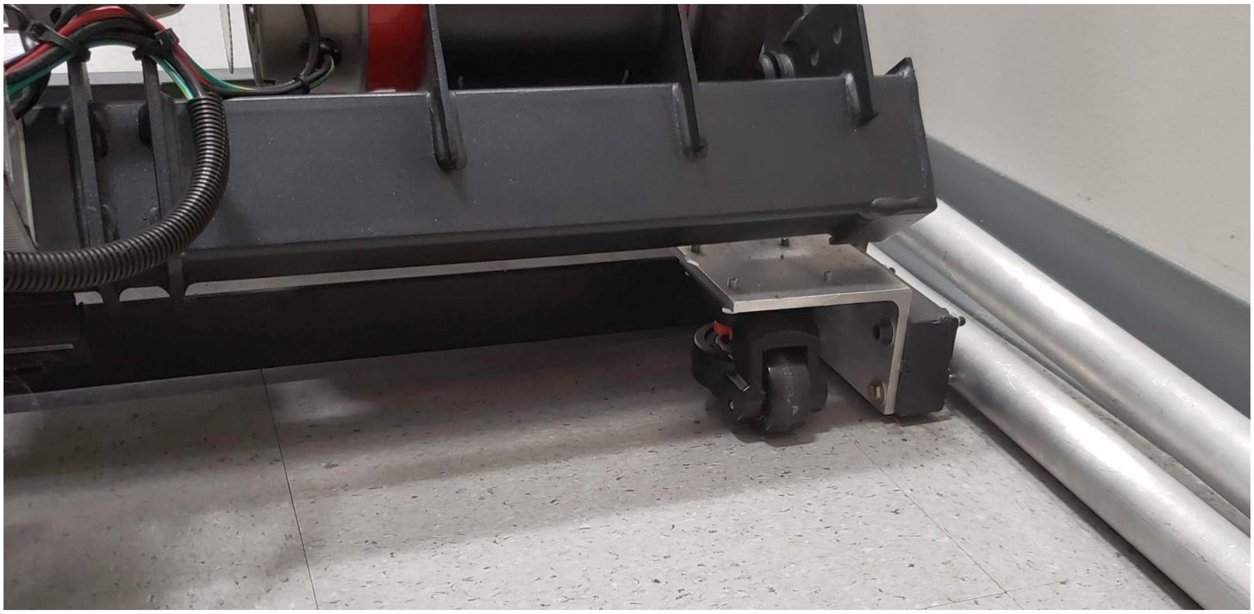

The stock treadmill deck folds up to reduce footprint size during storage. However, the support bar to hold the deck in the upright position was removed when the frame crossbars were cut. In order to retain the folding functionality, we installed locking hinges (McMaster #1570A122) on either side of the treadmill.

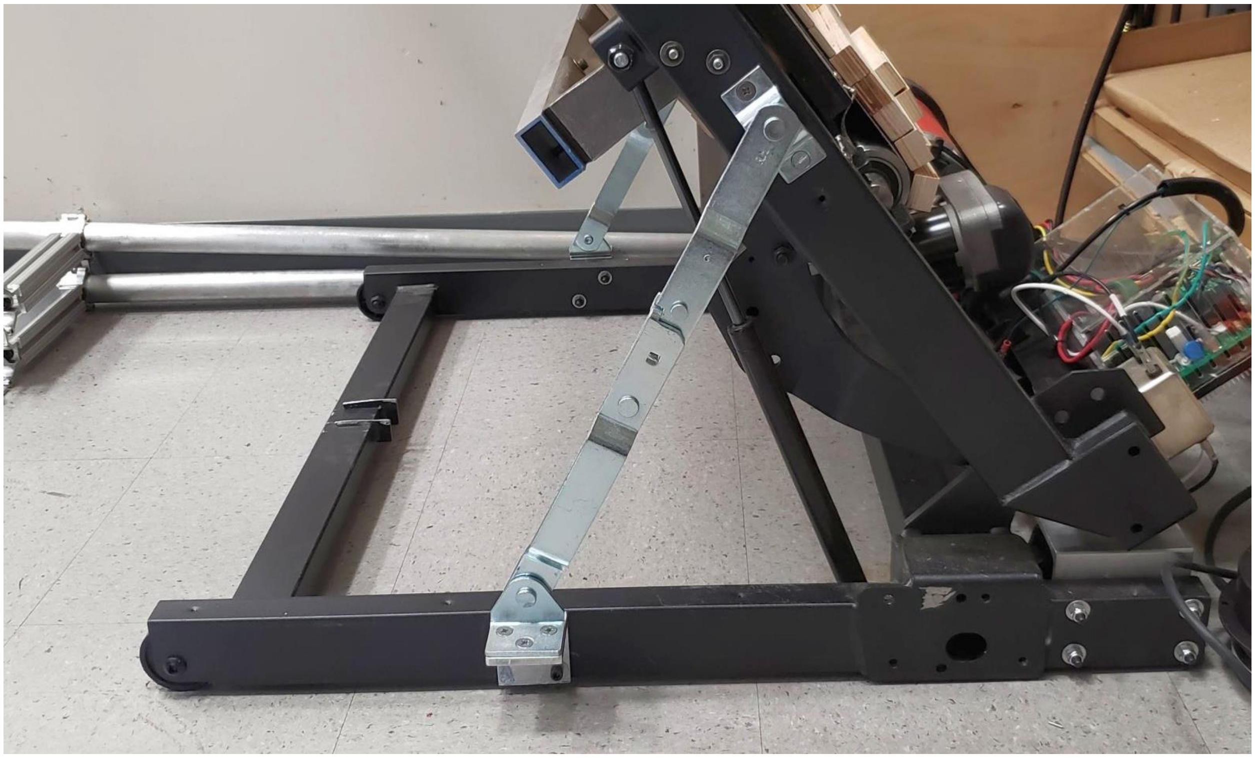

Supplement: Treadmill Modification Tips [file rsos201235supp2.pdf]
